# FetchM: streamlining genome and metadata integration for bacterial comparative genomics

**DOI:** 10.1093/bioadv/vbag124

**Published:** 2026-05-04

**Authors:** Tasnimul Arabi Anik, Peter Kjær Mackie Jensen, Anowara Begum

**Affiliations:** Environmental Microbiology Laboratory, Department of Microbiology, University of Dhaka, Dhaka 1000, Bangladesh; Department of Microbiology, Notre Dame University, Bangladesh, Dhaka 1000, Bangladesh; Department of International Health, Institute of Public Health, University of Copenhagen, Copenhagen N 2200, Denmark; Environmental Microbiology Laboratory, Department of Microbiology, University of Dhaka, Dhaka 1000, Bangladesh

## Abstract

**Motivation:**

Large-scale bacterial comparative genomics require comprehensive metadata describing genome assemblies and their biological context. While NCBI Genome holds the assemblies and BioSample stores key contextual fields such as collection date, host, and location, the lack of integration requires researchers to manually merge the two sources, slowing large-scale comparative studies. To address the gap, we built FetchM, a Python-based tool for automated retrieval, integration, analysis, and visualization of bacterial genome metadata from NCBI Genome and BioSample records. FetchM links genome assemblies to their corresponding BioSample entries via the NCBI Entrez API, appends missing contextual fields, and generates summary tables and plots. The tool supports sequence filtering by user-defined criteria (e.g. year, host, country, continent) and produces ready-to-analyze datasets for downstream workflows.

**Results:**

FetchM was applied to 14 382 *Vibrio cholerae* genome assemblies retrieved from NCBI, successfully integrating BioSample-associated metadata at scale. Temporal metadata were available for 89.07% of records, geographic metadata for 86.37%, and host metadata for 40.92%, highlighting persistent gaps in publicly available contextual data. Genome assembly sizes and annotation features were consistent with known biological expectations, supporting the accuracy of the retrieval workflow. Manual validation of selected records demonstrated high agreement between FetchM outputs and source metadata across key fields. These results indicate that FetchM enables reliable, scalable integration of genomic and contextual metadata, while also exposing limitations in metadata completeness across public repositories.

**Availability and implementation:**

FetchM is licensed under MIT and is freely available at https://github.com/Tasnimul-Arabi-Anik/FetchM and PyPI.

## 1 Introduction

Accurate and complete metadata associated with genomic datasets are critical for downstream bioinformatics analyses, comparative studies, and data reuse in genomic research. Public repositories such as the National Center for Biotechnology Information (NCBI) provide a wealth of microbial genomic data through platforms like BioSample and GenBank (Barrett *et al.* 2012). While GenBank contains data related to genome sequences, BioSample provides metadata describing the biological source of those genomes (Benson *et al.* 2018). Both types of information carry significant value for understanding the genomic context and drawing meaningful biological interpretations, especially in large-scale studies such as pangenome or comparative genomic analyses (Tettelin *et al.* 2008). However, retrieving and integrating both datasets can be time-consuming and technically challenging, especially when dealing with large genome collections.

NCBI provides tools that assist researchers in retrieving critical genomic and metadata (Kans 2023). Among them, the NCBI E-utilities (Entrez Utilities) is a powerful suite of programmatic tools that allows users to access and retrieve data from various NCBI databases, including GenBank, BioSample, and PubMed (Sayers *et al.* 2021, Kans 2023). These utilities support automated querying, downloading, and parsing of large-scale biological data via HTTP requests, streamlining data acquisition for bioinformatics workflows (Cock *et al.* 2009). However, retrieving biological source metadata for large bacterial genome datasets using existing tools can be time-consuming and often requires substantial manual effort. Integrating genomic data with associated metadata into a unified dataset would greatly facilitate downstream analyses and improve biological interpretation.

In this study, we developed FetchM (https://github.com/Tasnimul-Arabi-Anik/FetchM), a workflow that operates on standard NCBI bacterial genome dataset exports and automates the retrieval, integration, and partial standardization of genome-associated metadata, thereby reducing manual effort and enabling efficient large-scale analyses. We applied this approach to analyze metadata from 14 398 *Vibrio cholerae* genomes obtained from the NCBI Genome database. *V. cholerae* is a globally significant human pathogen and the causative agent of cholera, a severe diarrheal disease responsible for periodic pandemics and recurring outbreaks, particularly in low- and middle-income countries (LMICs) (Harris *et al.* 2012, Ojeda Rodriguez *et al.* 2025). Given its ecological adaptability and genetic diversity, *V. cholerae* has been isolated from a wide range of environments and hosts, making comprehensive metadata analysis essential for understanding its evolution, epidemiology, and global distribution. By systematically compiling and summarizing genome-associated metadata, including temporal, geographic, host, and assembly-related features, this study provides a comprehensive overview of publicly available *V. cholerae* genomes and showcases the ability of FetchM to efficiently retrieve and analyze large-scale microbial metadata from NCBI.

## 2 Methods

### 2.1 Implementation

FetchM v0.1.15 was implemented in Python (≥3.9) as a command-line workflow for retrieval, harmonization, summarization, and optional download of bacterial genome-associated metadata from NCBI. It takes as input an NCBI genome assembly list (ncbi_dataset.tsv) file from NCBI genome database (https://www.ncbi.nlm.nih.gov/datasets/genome/) for a target pathogen, which contains genome assembly identifiers but lacks detailed contextual metadata (e.g. collection date, geographic location, host). After loading the input table with pandas (v2.2.3), FetchM retrieves linked BioSample metadata from NCBI using HTTP requests implemented through “requests” (v2.32.3), with XML parsing performed by “xmltodict” (v0.14.2). The primary retrieval route uses BioSample E-utilities XML. When the primary BioSample XML record is incomplete, exposes no usable attributes, or resolves to an accession that does not match the queried BioSample identifier, FetchM attempts a secondary recovery path using NCBI E-utilities summary output to recover the BioSample-linked metadata. Metadata fields including collection date, geographic location, isolation source, and host are extracted from linked BioSample records and appended to the main assembly table.

**Figure 1:**
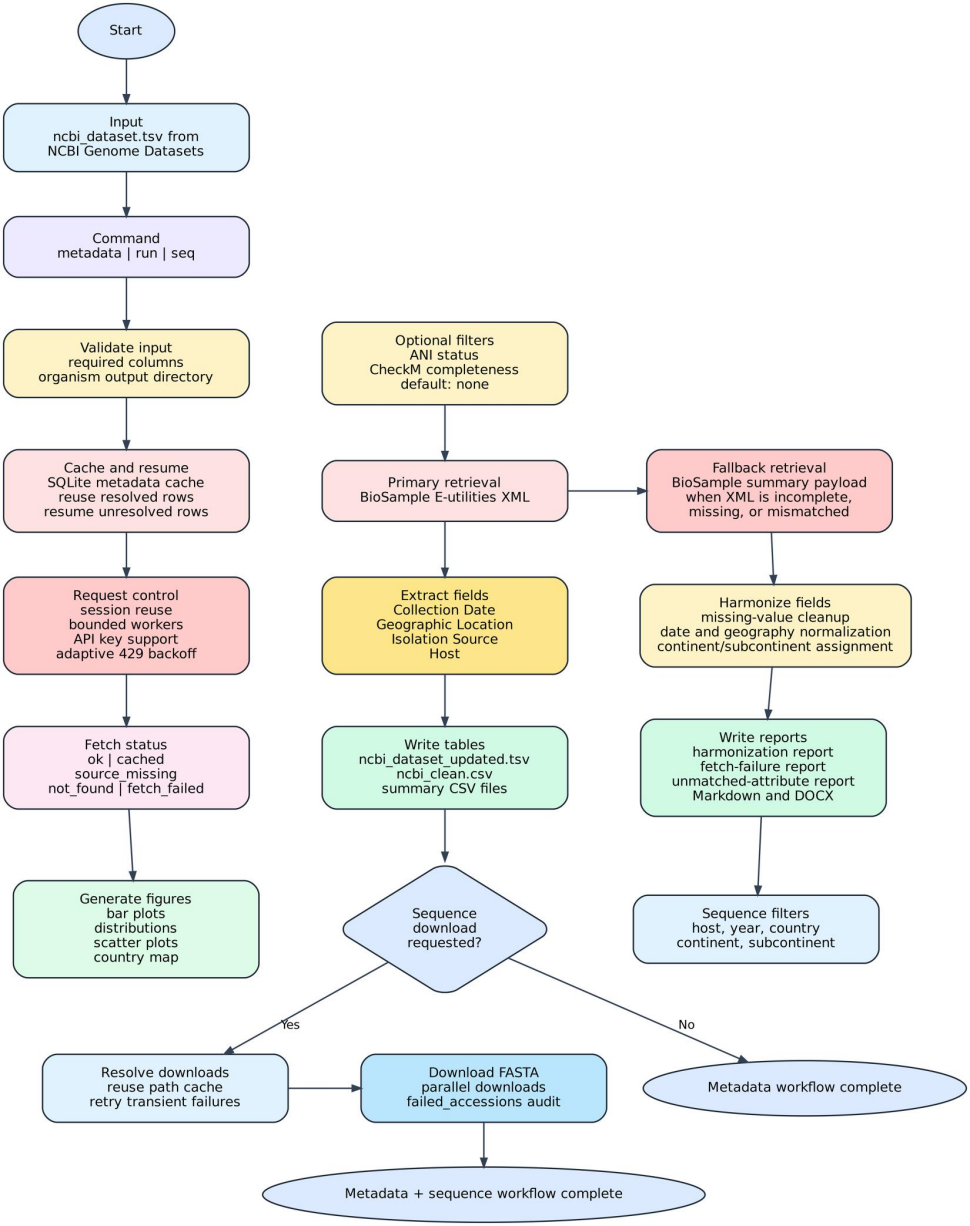
Overview of the FetchM workflow.

To improve robustness and performance, FetchM maintains persistent “requests.Session()” connections, supports optional NCBI API-key authentication, uses bounded concurrent metadata retrieval, and stores resolved BioSample metadata in a persistent SQLite cache within each organism-specific output directory. In the current release, metadata resume mode (“—resume-metadata”) allows reruns to reuse prior metadata results and refetch only unresolved rows. Retry and backoff strategies are applied to transient request failures, including rate limiting (“429” responses), dropped connections, and related recoverable request errors. The request interval is adjusted adaptively when throttling is encountered and is gradually relaxed after sustained successful requests. The overall workflow of FetchM is illustrated in Fig. 1.

### 2.2 Data processing and standardization

Core data processing is performed using pandas (v2.2.3). Numerical and statistical analyses are performed using SciPy (v1.15.1). Static visualizations are generated using Matplotlib (v3.10.1) and Seaborn (v0.13.2), whereas geographic map outputs are generated using Plotly (v6.6.0) with Kaleido (v1.2.0). Progress during large metadata runs is tracked using tqdm (v4.67.1), and the command-line interface is implemented using argparse (Python standard library). FetchM also generates tabular outputs, figures, and narrative report documents in Markdown and DOCX format using “python-docx” (v1.2.0). The cleaned metadata table (“ncbi_clean.csv”) is designed for direct downstream reuse in comparative genomics workflows, including tools such as PanR2 (https://github.com/Tasnimul-Arabi-Anik/PanR2), without additional reformatting.

FetchM supports optional filtering based on assembly-level quality and taxonomic validation metadata present in the NCBI input table. CheckM completeness filtering is disabled by default and can be enabled by the user when a minimum completeness threshold is required, acknowledging that completeness metadata are not uniformly available across all assemblies (Parks *et al.* 2015). ANI-based filtering is also optional; in the current release, all ANI statuses are retained by default unless explicitly restricted by the user (e.g. to entries with ANI status OK), allowing flexible control over taxonomic stringency (Jain *et al.* 2018). During processing, FetchM records the number of rows retained and removed by each active filter to ensure transparency and reproducibility.

After retrieval, the dataset is standardized with pandas (v2.2.3) to support consistent downstream analyses. Collection-date strings are reduced to four-digit year representations where a valid year can be recovered. Host and isolation-source values are harmonized primarily by normalizing missing-value expressions and cleaning inconsistent text formatting, while retaining source-derived labels where possible. FetchM does not perform full ontology-based normalization of all synonymous labels, and some variation may therefore remain in public-source metadata. Geographic location values are harmonized to a country-level representation where possible and are then used to assign continent and subcontinent categories. FetchM distinguishes between metadata-value classes and metadata-retrieval outcomes. The value “unknown” denotes cases where a field is semantically present but contains only missing-like or unknown-like content in the source metadata (e.g. “NA,” “missing,” “unknown,” or equivalent representations after harmonization). The value “absent” denotes cases where no usable value could be recovered for that field from the linked metadata sources. Retrieval outcomes are tracked separately using fetch-status classes (“ok,” “cached,” “source_missing,” “not_found,” and “fetch_failed”), allowing biologically missing metadata to be distinguished from unresolved or failed retrieval. To support interpretability and quality control, FetchM generates a metadata harmonization report, a metadata-fetch failure report, and an unmatched-attribute report summarizing unresolved records and source attributes that were encountered but not mapped.

### 2.3 Statistical analysis and visualization

FetchM generates summary statistics and visualizations from the harmonized dataset to support rapid assessment of metadata distributions. These include counts and frequency distributions for host, isolation source, geographic location, and collection year, together with summaries of assembly and annotation metrics. Static visualizations produced with Matplotlib (v3.10.1) and Seaborn (v0.13.2) include assembly-size distributions, annotation-feature distributions, and bar charts summarizing temporal and categorical metadata. A country-level geographic choropleth is generated using Plotly (v6.6.0) with Kaleido (v1.2.0). Scatter plots are used to assess relationships between collection year and selected genomic features; when too few valid observations are available, these plots are skipped automatically rather than interrupting the workflow. Correlation analyses are performed using Pearson’s and Spearman’s coefficients implemented in SciPy (v1.15.1). In the current workflow, assembly sequence length, total annotated gene count, and protein-coding gene count are evaluated against collection year after exclusion of records lacking usable temporal metadata.

### 2.4 Performance, reproducibility, and validation

FetchM is designed to operate reproducibly on standard workstation environments through explicit command-line parameters, persistent output files, cached metadata retrieval, and controlled request pacing. Retrieved BioSample metadata are stored in a SQLite cache within each organism-specific output directory, allowing repeated analyses of the same dataset without re-querying previously resolved accessions. In the current release, confirmed non-transient metadata outcomes, including successful retrievals and source-missing records, are retained in cache for reuse, whereas transient failures remain eligible for refetching in later runs. Resume mode (“—resume-metadata”) further reduces rerun time by reusing prior metadata outputs and refetching only unresolved rows.

Progress is tracked in real time using tqdm (v4.67.1). Benchmarking and validation were performed on an Ubuntu 24.04 LTS workstation (AMD Ryzen 7 CPU, 16 GB RAM, Python 3.12.8). As an end-to-end validation case study, FetchM v0.1.15 was applied to a *V. cholerae* genome dataset exported from NCBI Datasets. The workflow was executed under the current default metadata-retrieval settings, with optional API-assisted retrieval available for accelerated access to NCBI E-utilities. In a smaller regression test using the bundled “test.tsv” dataset, an initial metadata run completed successfully and a second run using “—resume-metadata” skipped metadata refetching and completed substantially faster, confirming the expected reuse behavior of the caching and resume implementation. Because NCBI metadata availability and response behavior can vary over time, exact metadata completeness and runtime may differ across datasets and run dates. All source code, example datasets, and usage instructions are available at: https://github.com/Tasnimul-Arabi-Anik/FetchM.

## 3 Results

### 3.1 Genome retrieval, assembly, and annotation features

**Figure 2:**
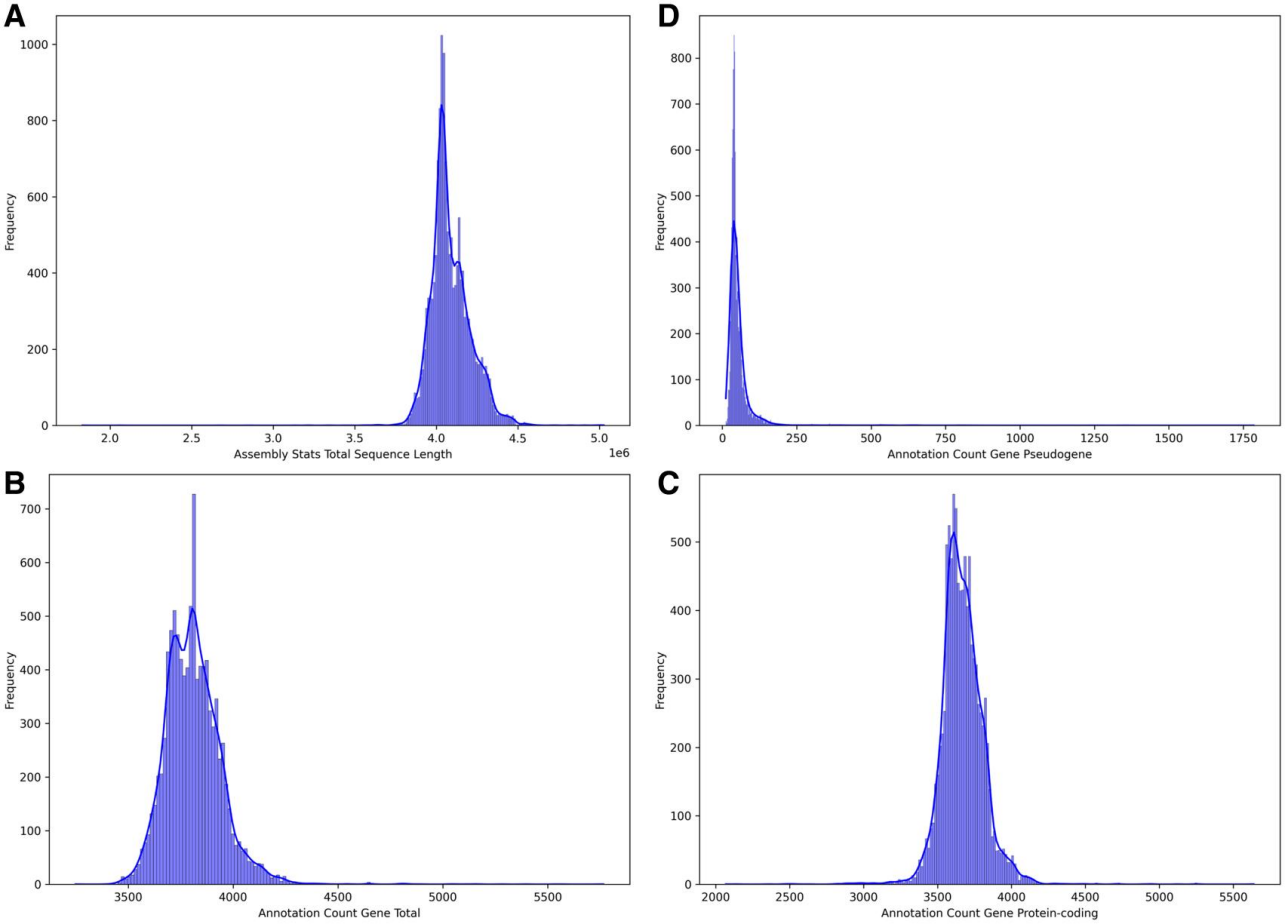
Summary of genome assembly and annotation statistics for *V. cholerae* genomes retrieved from NCBI. (A) Distribution of total genome sequence length (Mbp). (B) Distribution of total annotated gene counts (PGAP-annotated assemblies only). (C) Distribution of protein-coding gene counts. (D) Distribution of pseudogene counts across genomes.

As of 23 March 2026, a total of 14 398 *V. cholerae* genomes were available in the NCBI Genome database. Using FetchM, 14 382 genomes and their associated BioSample metadata were successfully retrieved and processed, with a small number excluded due to inconclusive or failed taxonomic status. The full dataset is available as File 1, available as supplementary data at *Bioinformatics Advances* online. The total sequence length ranged from 1.83 Mbp to 5.03 Mbp, with a mean of 4.09 Mbp and a median of 4.06 Mbp (Fig. 2A). Gene annotation metrics were assessed only for assemblies annotated via the NCBI Prokaryotic Genome Annotation Pipeline (PGAP). The total number of annotated genes per *V. cholerae* genome varied from 3249 to 5765, with a mean of 3811 and a median of 3803 (Fig. 2B). The number of protein-coding genes ranged from 2069 to 5638, with a mean of 3669 and a median of 3659, consistent with the expected coding potential of *V. cholerae* (Fig. 2C). In contrast, the number of pseudogenes exhibited greater variability, ranging from 12 to 1785 per genome (Fig. 2D). The mean pseudogene count was 57, while the median was 42, suggesting that most genomes contained relatively few pseudogenes, although a subset harbored a disproportionately high number.

### 3.2 Temporal and geographic metadata diversity

Temporal metadata (year of isolate collection) was available for 89.07% (*n = *12 810) of records, while 10.93% (*n = *1572) lacked this information. Isolate collection years ranged from 1934 to 2026, with the majority between 2001 and 2025. The peak occurred in 2018, accounting for 11.31% (*n = *1626) of all entries.

**Figure 3:**
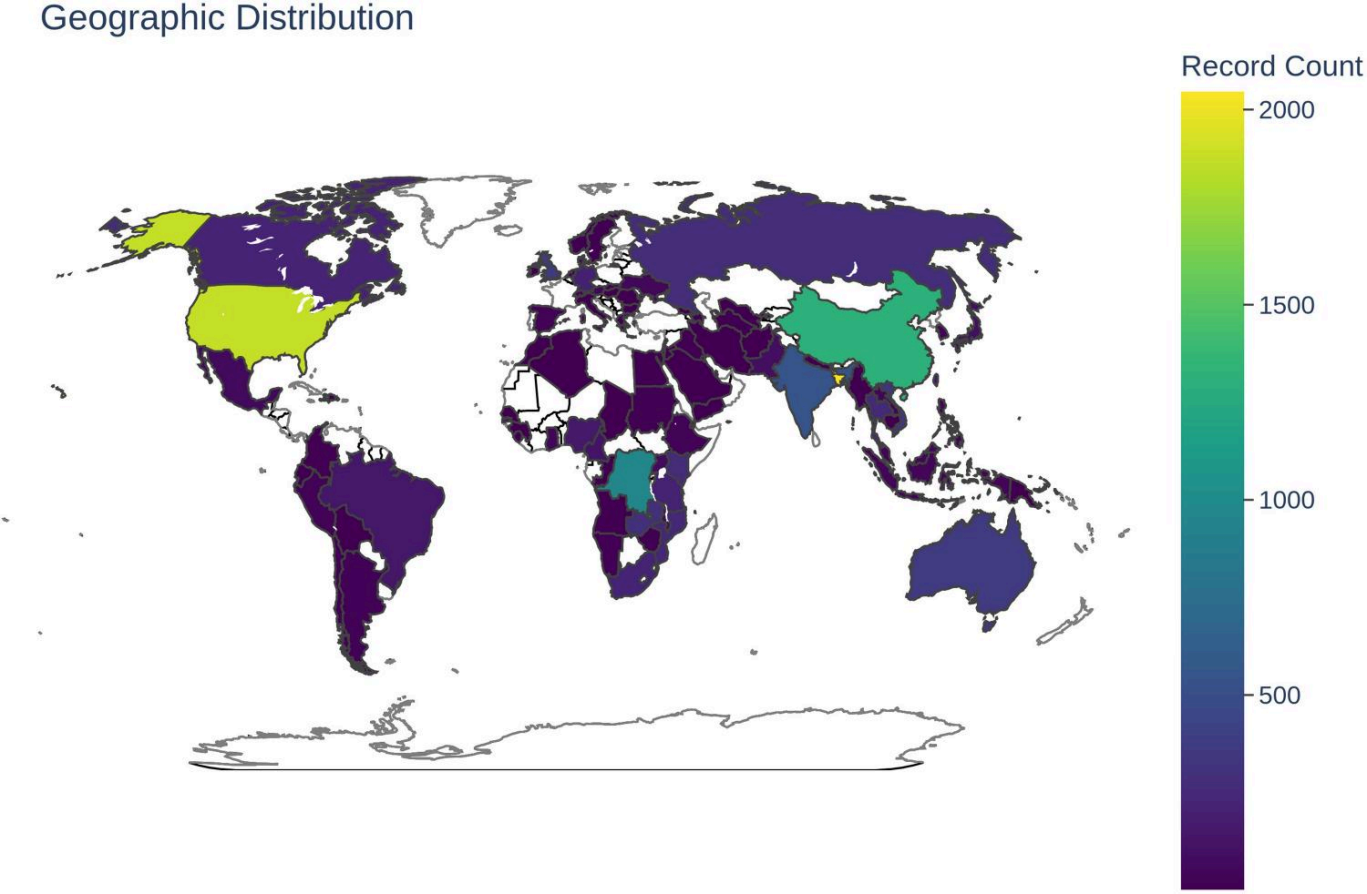
Geographic distribution of *V. cholerae* genomes by country. Bar plot showing the number and proportion of genomes contributed by each geographic location. A total of 95 unique locations were identified, with the highest contributions from Bangladesh, the United States, China, the Democratic Republic of the Congo, and Haiti.

**Figure 4:**
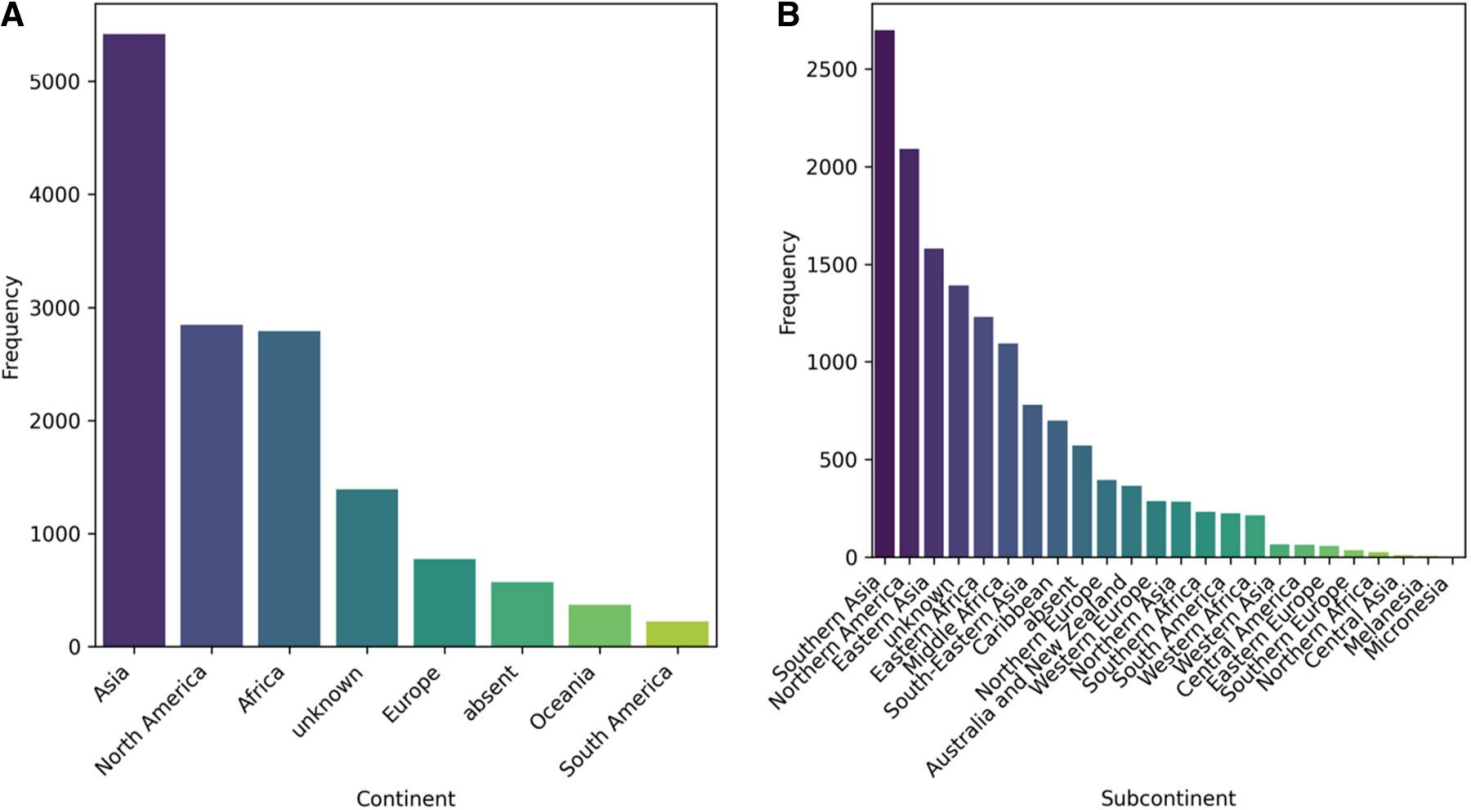
Geographic distribution of *V. cholerae* genomes at broader spatial scales. (A) Distribution of genomes across continents, showing Asia as the most represented region, followed by North America and Africa. (B) Distribution of genomes across subcontinents, highlighting Southern Asia, Northern America, and Eastern Asia as major contributors.

In addition to temporal trends, the geographic distribution of isolates was also evaluated. Metadata analysis revealed 95 unique geographic locations across the dataset (Fig. 3). The highest contributions for geographic location were Bangladesh (14.23%, *n = *2046), United States (13.04%, *n = *1876), China (9.15%, *n = *1316), Democratic Republic of the Congo (6.65%, *n = *956), and Haiti (4.83%, *n = *695). At the continent level, Asia (37.67%, *n = *5417) accounted for the highest representation, followed by North America (19.80%, *n = *2848), Africa (19.41%, *n = *2791), Europe (5.37%, *n = *772), and Oceania (2.57%, *n = *370) (Fig. 4A). South America had the lowest contribution among all continents. At the subcontinent level, Southern Asia (18.77%, *n = *2700) contributed the largest number of *V. cholerae* genome sequences, followed by Northern America (14.53%, *n = *2089), Eastern Asia (10.98%, *n = *1579), Eastern Africa (8.55%, *n = *1230), and Middle Africa (7.61%, *n = *1094) (Fig. 4B). Other subcontinents such as Central America, Northern Africa, and Melanesia reported relatively fewer sequences, which may reflect differences in genomic surveillance capacity or reporting practices. Geographic metadata remained incomplete for 13.63% (*n = *1960) of retained records, including 9.66% (*n = *1390) explicitly unknown values and 3.96% (*n = *570) values that were absent after metadata retrieval, highlighting persistent gaps in metadata completeness across the dataset.

### 3.3 Variability in host metadata

**Figure 5:**
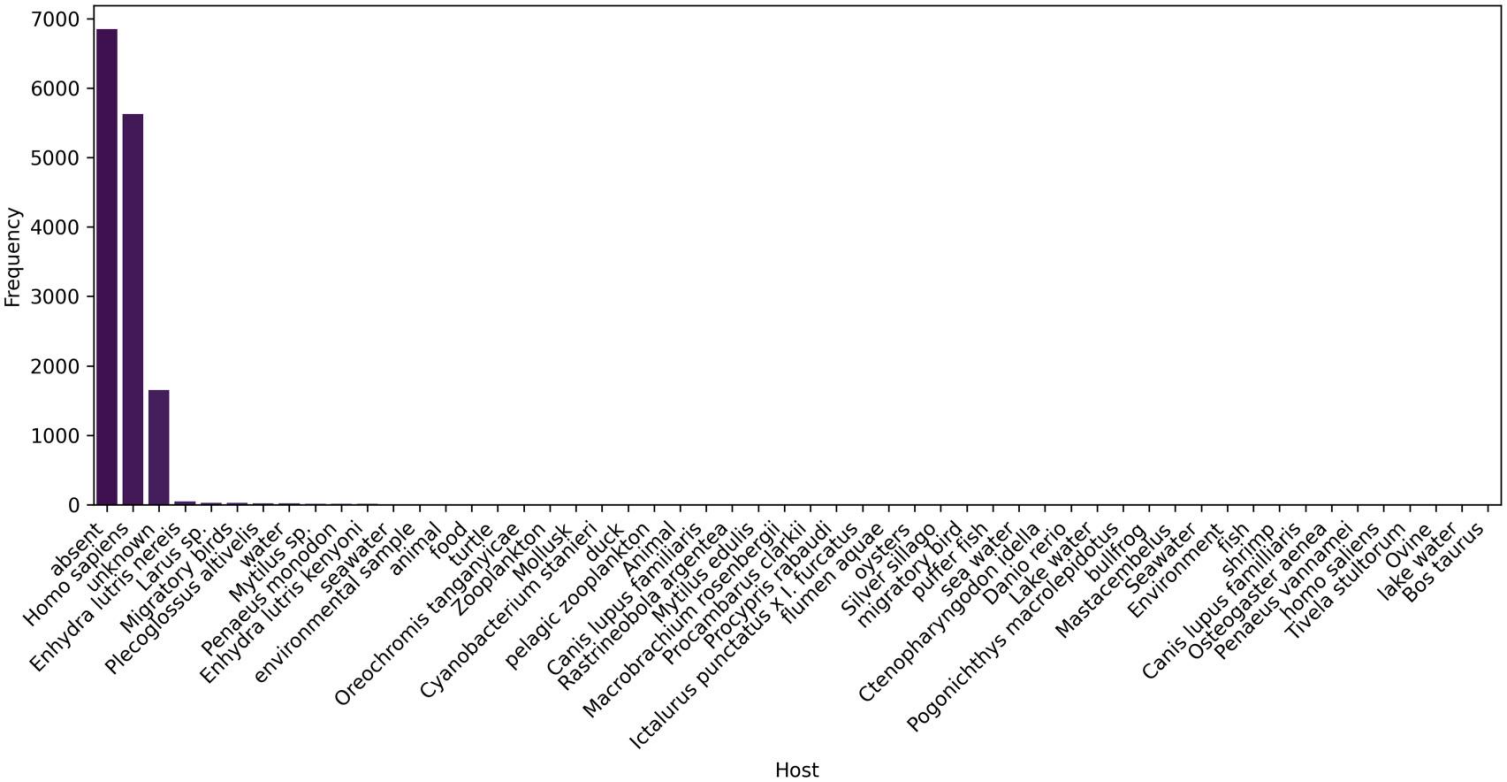
Distribution of host annotations for *V. cholerae* genomes. Bar plot showing the frequency and proportion of host categories among genomes with available host metadata. The majority of annotated hosts correspond to *Homo sapiens*, with smaller contributions from other hosts such as *Enhydra lutris nereis*, *Larus* sp., migratory birds, and *Plecoglossus altivelis*.

Host metadata exhibited greater variability compared to other metadata fields. Host information was available for 40.92% (*n = *5885) of retained genome entries, while 11.47% (*n = *1649) were explicitly recorded as unknown and 47.62% (*n = *6848) were absent after metadata retrieval. This distribution may reflect differences in reporting practices across public databases, as well as the inclusion of environmental samples where a host may not be applicable. Among entries with available host information, the most frequent annotations were *Homo sapiens* (39.13%, *n = *5627), followed by *Enhydra lutris nereis* (0.33%, *n = *48), *Larus* sp. (0.19%, *n = *28), migratory birds (0.17%, *n = *24), and *Plecoglossus altivelis* (0.12%, *n = *17) (Fig. 5). Other host annotations were rare and often heterogeneous in format. For example, some entries describe environmental contexts broadly (e.g. “water”), while others provide more specific terms (e.g. “seawater”), despite referring to similar sample origins. Similarly, biological host entries vary in specificity, ranging from precise scientific nomenclature (e.g. *Bos taurus*) to more general or missing descriptors (e.g. “animal” or empty fields).

### 3.4 Temporal trends in genome assembly length and annotation features

**Figure 6:**
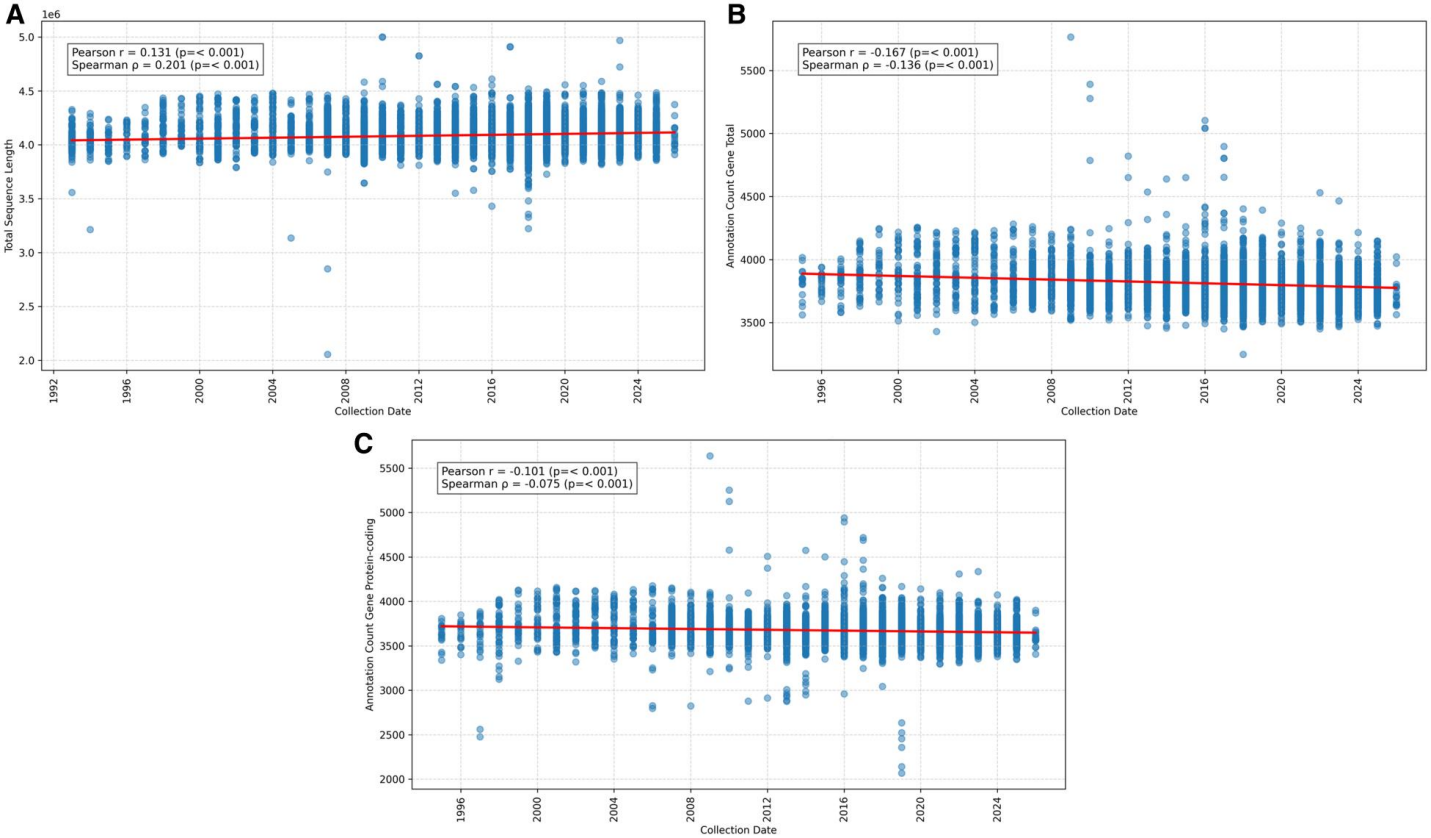
Temporal trends in genome assembly and annotation metrics for *Vibrio cholerae*. (A) Relationship between collection year and genome assembly length (Mbp), showing a weak positive correlation. (B) Relationship between collection year and total annotated gene count, indicating a slight decreasing trend. (C) Relationship between collection year and protein-coding gene count, also showing a marginal decline.

All *V. cholerae* genomes were analyzed to assess potential temporal trends in assembly length and gene annotation statistics, using PGAP as the sole annotation pipeline to avoid methodological variability. Assembly sequence length remained relatively stable over time, averaging between ∼4.0 and 4.5 Mb, with Pearson’s *r *= 0.131 (*P < .*001) and Spearman’s ρ  =  0.201 (*P < .*001) (Fig. 6A), indicating a weak but statistically significant positive correlation and suggesting no strong biological trend. Minor fluctuations may reflect differences in assembly methods used by various researchers. Total annotated gene counts showed a slight decreasing trend (*r *= −0.167, *P < .*001; ρ = −0.136, *P < .*001) (Fig. 6B). Similarly, protein-coding gene counts also declined marginally (*r *= −0.101, *P < .*001; ρ = −0.075, *P < .*001) (Fig. 6C). All analyses were performed on filtered datasets after outlier removal, resulting in 11 870 usable records for assembly length and 8174 records for gene-based analyses.

### 3.5 Validation of FetchM

Processing of the *V. cholerae* dataset (14 382 genomes) involved metadata retrieval and analysis, with a total runtime of approximately 1 hour 12 minutes. The overall processing time may vary depending on network bandwidth, use of an NCBI API key, server response times, and system performance. Prior to large-scale analysis, FetchM was validated using a test dataset (https://github.com/Tasnimul-Arabi-Anik/FetchM/blob/main/test.tsv), where processing 100 genomes required approximately 40 seconds, demonstrating efficient performance on smaller datasets. While metadata retrieval from the test dataset was accurate, its limited size was insufficient for comprehensive validation of FetchM. Therefore, a subset (*n = *200) of records from the *V. cholerae* dataset was manually checked for each metadata field to evaluate the accuracy of retrieval and classification (Table 1). For the field “collection date or year,” FetchM showed complete agreement with manual checking, correctly identifying all 177 records with available metadata and all 23 records lacking such information, with no false positives or false negatives observed. A separate validation of 200 records labeled as missing or unknown confirmed that entries explicitly annotated with terms such as “unknown,” “not collected,” or similar variants were consistently classified as unknown, while entries lacking any retrievable information were correctly labeled as absent.

**Table 1:** Validation of FetchM metadata retrieval and classification against manual curation.^a^

Metadata column	Manual check	FetchM (present)	FetchM (absent/unknown)	Total
Collection date/year	Present	177	0	200
	Absent	0	23	
Geographic location	Present	189	1	200
	Absent	0	10	
Host	Present	88	0	200
	Absent	0	112	
Isolation source	Present	163	0	200
	Absent	0	37	

a Manual validation was performed on a subset of 200 randomly selected records for each metadata field. “Present” indicates that metadata was correctly retrieved by FetchM, while “Absent/Unknown” indicates that metadata was either not available or explicitly labeled as missing. Values represent counts of records in each category. Minor discrepancies (e.g. geographic location) were due to variation in field naming and were resolved in subsequent updates.

For geographic location, FetchM showed near-complete agreement with manual annotations, correctly identifying 189 records with available metadata and all 10 records lacking such information. One record was initially missed due to variation in variable naming but was resolved following a tool update. Validation of 200 missing or absent records confirmed correct classification. Approximately 0.98% of geographic entries were initially not assigned due to variation in naming formats. Following refinement of the parsing logic, these cases were resolved in the evaluated dataset. However, for previously unseen or highly irregular location strings in user-submitted metadata, some values may still remain unassigned. For host annotation, FetchM showed complete agreement, correctly identifying all 88 records with available host information and all 112 records lacking such information, with no misclassification. Similarly, validation of 200 missing or unknown records confirmed accurate classification. For isolation source, FetchM demonstrated complete agreement, correctly identifying all 163 records with available metadata and all 37 records lacking such information. Validation of 200 missing or absent records confirmed consistent classification.

## 4 Discussion

FetchM addresses key challenges in large-scale microbial genomics by providing an integrated framework for automated metadata retrieval, harmonization, quality control, and visualization. Unlike existing approaches such as EDirect, which require manual query construction and scripting (Sayers *et al.* 2021), FetchM enables end-to-end processing starting from standard NCBI genome dataset exports. By programmatically linking genome assemblies to BioSample records, the tool supplements incomplete metadata fields, including collection date, geographic origin, host, and isolation source, thereby substantially reducing manual effort and potential user error. A major strength of FetchM lies in its usability and workflow integration. The tool combines metadata retrieval, filtering, and visualization within a single command-line pipeline, eliminating the need for multiple external tools or custom scripts (e.g. ggplot2-based workflows). In addition, FetchM generates publication-ready outputs without requiring advanced programming expertise, making it accessible to a broader range of researchers, including those with limited bioinformatics backgrounds.

From a performance perspective, FetchM demonstrates efficient scalability for large genomic datasets. In this study, processing 14,382 *V. cholerae* genomes required approximately 1 hour and 12 minutes on a standard workstation, while smaller test datasets were processed within seconds. The use of persistent HTTP sessions, concurrent metadata retrieval, and SQLite-based caching significantly improves runtime efficiency, particularly for repeated analyses. The resume functionality further enhances reproducibility and reduces computational overhead by avoiding redundant metadata retrieval. Validation results further support the reliability of FetchM. Manual verification of selected records demonstrated near-complete agreement between retrieved metadata and source BioSample records across multiple fields, including temporal, geographic, host, and isolation source metadata. Minor discrepancies were primarily associated with variations in source metadata naming conventions rather than errors in the retrieval workflow itself. These findings indicate that FetchM provides a robust and accurate framework for metadata integration, while also highlighting the inherent limitations of publicly available datasets. Beyond technical performance, FetchM has broad applicability across multiple areas of microbial genomics. The ability to generate curated, analysis-ready datasets enables downstream applications such as phylogeographic studies, host–pathogen interaction analysis, and temporal trend assessment. The integrated filtering options further allow users to tailor datasets based on criteria such as host, geographic region, or collection year, supporting flexible and reproducible study design.

In addition to demonstrating tool performance, the application of FetchM to the *V. cholerae* dataset provides insights into genome characteristics and metadata distribution. The observed genome size distribution closely matched the expected range for *V. cholerae* (4.0–4.1 Mbp) (Heidelberg *et al.* 2000). Assemblies with markedly shorter lengths likely represent incomplete or fragmented genomes. Since quality-based filtering (e.g. using CheckM) was not applied, the dataset includes genomes with varying levels of assembly completeness, which may contribute to the observed variation in genome length. The gene annotation metrics were assessed using only genome assemblies annotated via the NCBI PGAP to ensure consistency and accuracy across records. Pseudogene counts varied widely across genomes, which may reflect both biological processes such as niche adaptation and technical factors including sequencing platform, assembler choice, and coverage depth (Gil and Latorre 2012, Cooley and Wright 2024). These observations highlight the importance of considering both biological and methodological factors when interpreting large genomic datasets.

Temporal metadata (year of isolate collection) was available for 89.07% of records, indicating relatively high completeness. The large number of *V. cholerae* genome sequences recorded in 2018 may reflect several cholera outbreaks that occurred across different countries during that year, prompting intensified surveillance and sequencing efforts. In Somalia, a prolonged outbreak that began in late 2017 continued throughout 2018, with over 6600 suspected cases and 45 deaths reported by year’s end, and laboratory confirmation of *V. cholerae* O1 Ogawa in multiple stool samples (WHO 2018). Algeria experienced its first cholera outbreak in over 20 years, with 291 suspected cases reported between August and September 2018, caused by *V. cholerae* O1 El Tor, Ogawa (Cherifi *et al.* 2022). In Zimbabwe, a major epidemic erupted in September 2018, leading to over 10 700 suspected cases and 371 confirmed infections by early 2019 (WHO 2019). These outbreaks likely led to increased sample collection and sequencing activities as part of public health response and molecular epidemiology efforts, contributing to the observed spike in genome data for that year. Interestingly, the dataset also includes historically significant records dating as far back as 1934, alongside very recent genomes collected in 2026. This broad temporal range would allow researchers to perform longitudinal analyses and study evolutionary trends over nearly nine decades.

Geographically, the dataset reflects contributions from both endemic and non-endemic regions. High-burden countries such as Bangladesh, the Democratic Republic of the Congo (DRC), Haiti, and Zambia are well represented due to ongoing outbreaks and public health surveillance efforts. Conversely, the United States contributes a large number of genomes despite low endemicity, owing to strong laboratory-based genomic surveillance programmes (CDC 2019). Bangladesh remains one of the world’s cholera hotspots, with consistently high disease prevalence; nationwide surveillance between 2014 and 2018 found that 6.2% of diarrhea patients tested positive for *V. cholerae* O1, with persistent biannual seasonal peaks in regions such as Dhaka and Chittagong (Islam *et al.* 2020). China’s high representation likely results from its public health laboratories and large-scale sequencing initiatives, while the DRC reflects ongoing efforts to investigate epidemics in the Great Lakes region, including outbreaks between 2009 and 2012 (Kule *et al.* 2022). Haiti continues to contribute genomic data following its major outbreak beginning in 2010, where genomic epidemiology traced the source to South Asia (Katz *et al.* 2013). Zambia has also experienced sporadic outbreaks that have been monitored through both epidemiological and genomic surveillance efforts (Sinyange *et al.* 2018). The high representation of sequences from these countries reflects the dual influences of surveillance capacity and disease burden, underscoring the epidemiological importance of sustained genomic monitoring in cholera-endemic regions.

Host metadata completeness was the lowest among all categories. This lower completeness is influenced by both biological and reporting factors. For example, environmental samples (e.g. water) may not have an associated host, and in some cases host information is explicitly labeled as missing. In such situations, the isolation source may still provide useful information and can sometimes serve as a proxy for host (e.g. mussels). Therefore, lower completeness in host metadata does not necessarily indicate a lack of biological relevance for all downstream analyses. However, even when host metadata is present, variability in terminology (e.g. “human” vs. *Homo sapiens*) can limit its usage for systematic analyses. While FetchM harmonizes missing data labels and standardizes fields such as collection date and geographic location, the wide variability in host annotations, particularly synonymous or inconsistently formatted labels, remains challenging to fully resolve. This limitation arises primarily from the heterogeneity of user-submitted metadata. These factors contribute to apparent metadata gaps despite accurate retrieval and highlight the challenges posed by inconsistent or ambiguous terminology. The lack of uniformity presents a significant obstacle for metadata-driven downstream analyses, such as ecological association studies or host–pathogen interaction mapping, which rely on structured and standardized data. As there is currently no universal standard for host annotation across public repositories, retrieving and interpreting host metadata remains one of the more challenging aspects of large-scale genomic datasets. Future updates of FetchM will aim to further improve normalization of host-related fields to address these limitations.

We also analyzed annotation features over time using FetchM. Despite slight declines in annotated gene counts over time, the genomic stability of *V. cholerae* remains evident, with no substantial long-term trends in genome size. Given the consistent annotation method (PGAP), these trends are unlikely to reflect methodological bias in gene calling, and instead may relate to differences in assembly completeness or sequencing data quality over time. Overall, the results highlight the genomic stability of *V. cholerae* across decades of global sampling.

Despite its strengths, FetchM is inherently dependent on the quality and consistency of publicly available metadata. Variability in field naming, incomplete records, and inconsistent reporting standards can affect downstream analyses, even when metadata retrieval is accurate. While the tool mitigates some of these challenges through harmonization and classification strategies, complete resolution will require improvements at the data submission level. In addition, FetchM relies on NCBI APIs for metadata retrieval, and its performance is therefore dependent on NCBI server behavior. In some cases, large-scale or taxon-specific queries (e.g. certain bacterial groups) may experience instability or incomplete responses during bulk retrieval. Such limitations are external to FetchM and may affect data completeness despite built-in retry and caching mechanisms. While not all limitations can be fully addressed within the current framework, future development of FetchM will focus on enhanced normalization of heterogeneous metadata fields and improved handling of non-standard annotations. Overall, FetchM provides a scalable, reproducible, and user-friendly solution for integrating genomic and contextual metadata, addressing a critical bottleneck in microbial comparative genomics while highlighting the continued need for improved metadata quality in public repositories.

## 5 Conclusion

In this study, we present FetchM as a scalable and reproducible framework for automated retrieval, integration, and analysis of genome-associated metadata from NCBI Genome and BioSample records. By combining metadata extraction, harmonization, filtering, and visualization within a single workflow, FetchM substantially reduces the manual effort required for large-scale comparative genomic analyses. The tool enables users to generate analysis-ready datasets and publication-quality outputs while maintaining flexibility through user-defined filtering and reproducible workflows. Validation against source BioSample records demonstrates that FetchM achieves high accuracy in metadata retrieval and classification across key fields, supporting its reliability for large-scale applications. In addition, performance benchmarking shows that the tool can efficiently process thousands of genomes within practical timeframes, aided by caching, concurrent retrieval, and resume functionality. These features collectively distinguish FetchM from existing approaches that rely on fragmented workflows or manual scripting. Application of FetchM to a large *V. cholerae* dataset highlights both the utility of the tool and the current limitations of publicly available metadata, particularly in host and contextual annotations. While FetchM mitigates many of these challenges through harmonization and structured outputs, the overall quality of downstream analyses remains dependent on the completeness and consistency of submitted metadata. Future development will focus on improving normalization of heterogeneous metadata fields. By enabling efficient and reliable integration of genomic and contextual data, FetchM provides a practical solution for accelerating microbial comparative genomics and supports more comprehensive investigations into pathogen evolution, epidemiology, and global distribution. FetchM is available at https://github.com/Tasnimul-Arabi-Anik/FetchM for adoption, validation, and further development.

## Supplementary Material

vbag124_Supplementary_Data

## Data Availability

FetchM is available as open-source software at GitHub (https://github.com/Tasnimul-Arabi-Anik/FetchM) and as a Python package on PyPI (https://pypi.org/project/fetchm/). The repository includes the source code, installation instructions, workflow documentation, and example input datasets, including test.tsv and vibrio_v2.tsv, which were used for testing and validation.
